# Cerebral Venous Sinus Thrombosis Secondary to Neurobrucellosis: A Case Report

**DOI:** 10.7759/cureus.32677

**Published:** 2022-12-19

**Authors:** Faisal Alattas, Osama Khojah, Abdulmalik Mukhtar, Rayan Khan, Maan Jamjoom, Aisha Halawani, Seraj Makkawi

**Affiliations:** 1 College of Medicine, King Saud Bin Abdulaziz University for Health Sciences, Jeddah, SAU; 2 Research and Development, King Abdullah International Medical Research Center, Jeddah, SAU; 3 Medicine, Ministry of the National Guard - Health Affairs, Jeddah, SAU; 4 Emergency Medicine, King Abdullah International Medical Research Center, Jeddah, SAU; 5 College of Applied Medical Sciences, King Saud Bin Abdulaziz University for Health Sciences, Jeddah, SAU; 6 Emergency Medicine, Ministry of National Guard-Health Affairs, Jeddah, SAU; 7 Medical Imaging, King Abdullah International Medical Research Center, Jeddah, SAU; 8 Medical Imaging, Ministry of National Guard - Health Affairs, Jeddah, SAU

**Keywords:** s-cerebral venous thrombosis, stroke, brucellosis complications, cerebral venous sinus thrombosis (cvst), neurobrucellosis, brucellosis

## Abstract

Brucellosis is a common infection that rarely causes cerebral venous sinus thrombosis (CVST). In this case, a 23-year-old male presented to the emergency department with status epilepticus. With a past medical history of drinking unpasteurized camel milk, elevated inflammatory markers, and evidence of brucellosis in the serum, the patient was diagnosed with brucellosis. Further investigations revealed left transverse sinus thrombosis extending to the jugular vein. The patient was treated with enoxaparin and a combination of doxycycline, ceftriaxone, and trimethoprim-sulfamethoxazole. This regimen led to rapid and significant clinical improvement in the signs and symptoms of the patient. CVST is a rare complication of neurobrucellosis that might present with signs and symptoms of meningitis. This case report highlights the importance of keeping neurobrucellosis as a possible cause of CVST in patients living in an area endemic to brucellosis.

## Introduction

Brucellosis is a bacterial zoonotic infection that is endemic to many developing countries [1]. The pathogen is transmitted to humans through direct contact with infected animals or indirectly through the consumption of unpasteurized milk or dairy products [1]. The Kingdom of Saudi Arabia has a higher incidence of brucellosis compared to the global average, with a reported average incidence of 15.34 per 100,000 annually from 2013 to 2018 [2]. Brucellosis is a multisystemic disease that has broad clinical manifestations. Clinical presentations can range from asymptomatic to severe illness depending on the organ involved [3]. The main clinical features of brucellosis include undulant fever, fatigue, weakness, myalgia, and arthralgia [4]. Furthermore, brucellosis’ neurologic involvement affecting the central and peripheral nervous systems has been reported in 2-7% of cases. The most common neurological manifestations are meningitis, meningoencephalitis, headache, confusion, elevated intracranial pressure, cranial neuropathies, and peripheral neuropathies [3,4]. Cerebral venous sinus thrombosis (CVST) is a rare disease with 1.16 to 2.02 cases per 100,000 globally, a female-to-male ratio of 3:1, and a median age of 37 [5]. CVST is a rare type of stroke that accounts for 0.5-3% of all stroke types [6]. Seizures, encephalopathies, and focal syndromes are among the symptoms [5]. In this case report, we describe a patient diagnosed with CVST secondary to neurobrucellosis presenting to the emergency department with status epilepticus.

## Case presentation

**Figure 1:**
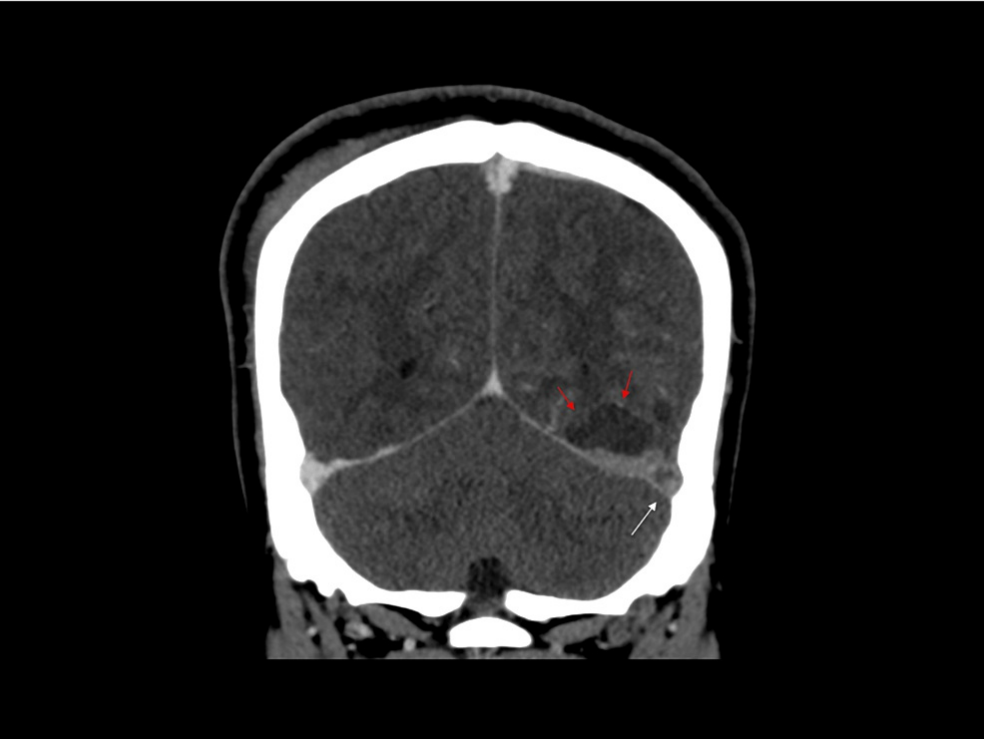
Contrast-enhanced computed tomography venogram White arrow: shows a filling defect at the left transverse sinus denoting thrombus; red arrows: associated left occipital lobe hypodensity in keeping with the infarction

**Figure 2:**
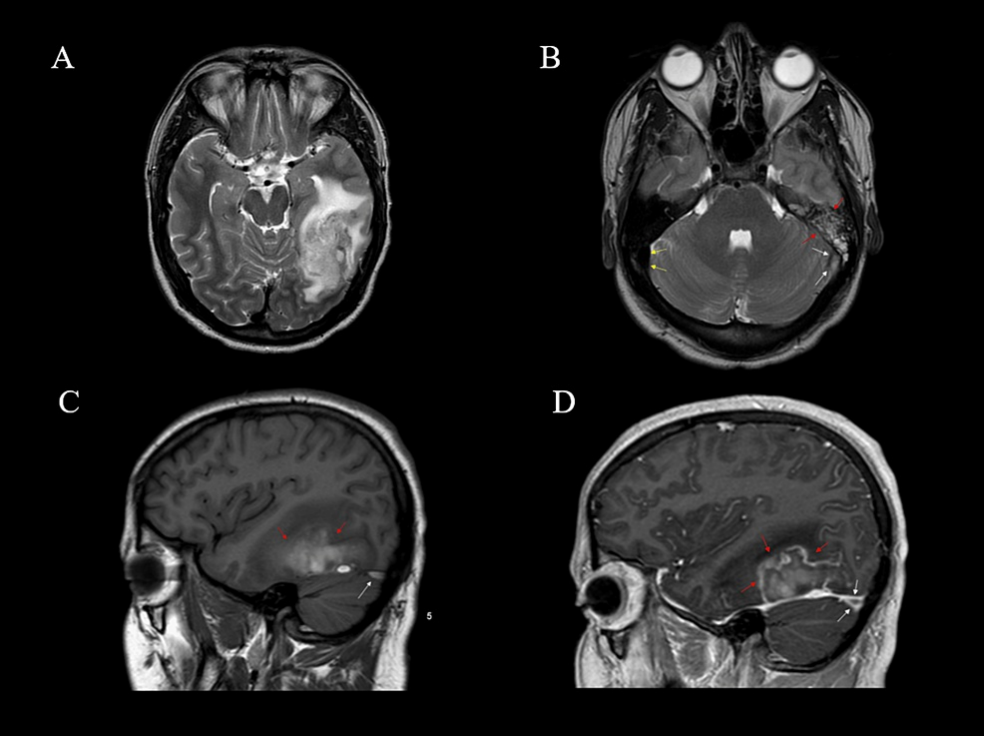
Brain magnetic resonance imaging (A) Axial T2WI: shows the extent of the venous infarction within the left occipital lobe. (B) Axial T2WI: white arrows show loss of flow voids at the distal left transverse sinus related to the thrombus, and red arrows show an associated left mastoid air cells effusion. On the other hand, yellow arrows show the normal flow void at the distal right transverse sinus. (C) Sagittal T1WI pre-gadolinium: white arrow shows T1 hyper-intense thrombus within the left transverse sinus, and red arrows show within the left occipital lobe infarction in keeping with the hemorrhagic nature of the infarction. (D) Sagittal T1WI post IV gadolinium injection: white arrows show an intraluminal filling defect within the left transverse sinus consistent with acute venous sinus thrombosis, and red arrows show a left occipital lobe hemorrhagic infarction with minimal irregular peripheral enhancement.

A 23-year-old male was brought to the emergency department after a first-time seizure. Three episodes of generalized tonic-clonic seizures were associated with a 10-day history of headache and subjective fever. These seizures were terminated only after placing him under sedation using propofol and midazolam. Past medical history was remarkable for long-term consumption of unpasteurized camel milk. The examination was remarkable for fever (38.0 °C). Investigations showed evidence of infection, which included high serum white blood cell count (WBC) 38.4 × 109/L, elevated erythrocyte sedimentation rate (ESR) 22 mm/hr, C-reactive protein (CRP) 58.5 mg/L (normal range; 0-5 mg/L), and lactic acid 33.03 mmol/L (normal range; 0.51-2.1 mmol/L). The results of the coagulation profile were unremarkable. Brain computed tomography (CT) without contrast showed a hyperdensity within the left transverse venous sinus. Prompt cerebral venography CT showed filling defects in the torcula, left transverse venous sinus, small jugular bulb, and proximal internal jugular vein (Figure 1). These findings were later recuperated by brain magnetic resonance imaging (MRI) (Figure 2). Hence, the patient was diagnosed with CVST and was started on a therapeutic dose of unfractionated heparin. Cerebral spinal fluid (CSF) analysis showed mild lymphocytic pleocytosis (6 leukocytes/ µL), elevated red blood cell (RBC) count (6240 cells/ µL) likely to be traumatic, and elevated glucose (7.20 mmol/L) and protein levels (1.24 g/L). Further CSF analysis using polymerase chain reaction was negative for human parechovirus, herpes simplex virus-1, herpes simplex virus-2, human herpes virus-6, Listeria monocytogenes, Varicella zoster virus, Escherichia coli K1, Haemophilus influenzae, Neisseria meningitidis, Streptococcus agalactiae, Enterovirus, and Cryptococcus neoformans/gattii. Although the CSF culture showed no growth that was likely because antibiotics were started prior to the lumbar puncture, and blood cultures were positive for brucella species. Immunoglobulin M (IgM) and IgG brucella antibodies were both present in the serum. Therefore, the patient was started on sulfamethoxazole/trimethoprim, doxycycline, and ceftriaxone. Two days later, the patient was extubated and his neurological examination was unremarkable. His hospital stay was lengthened by complications including a pulmonary embolism. Nevertheless, the patient’s fever and seizures completely resolved on this regimen, and the patient was discharged 14 days after presentation. CSF analysis was repeated and was found to be trending downward, as the white blood cell (WBC) count was 4 leukocytes/ µL, RBC count was 0 cells/ µL, protein level was 1.04 g/L, and glucose was 4.10 mmol/L. He was discharged on the aforementioned antibiotic regimen for three months in addition to warfarin for one year. Serial examinations and CSF analyses four weeks from discharge showed no recurrence of signs and symptoms and normalization of CSF results. CSF analysis showed a WBC count of 2 leukocytes/µL, RBC count of 310 cells/µL, protein levels of 0.23 g/L, and glucose of 3.20 mmol/L. The aforementioned extensive CSF analysis was repeated and was negative. Follow-up CT brain almost a year later showed partial recanalization of the transverse sinus. On his most recent appointment 15 months after his initial presentation, he was seizure free with a modified Rankin score of zero.

## Discussion

In 1999, Zaidan and Al Tahan published the first case of CVST secondary to neurobrucellosis after the patient presented with signs and symptoms of meningitis [7]. Since then, six more articles have been published describing patients with the same diagnosis [3,4,8-11]. CVST caused by neurobrucellosis is a rare presentation with good outcomes. This is the second known case describing a patient from Saudi Arabia. In many Saudi regions, drinking unpasteurized camel milk, a source of brucellosis, is not only the traditional way but the only acceptable way to consume it [12]. This has increased the burden of the disease in the region and made brucellosis one of the top differential diagnoses in many cases. Brucellosis-related cerebral vasculopathy is uncommon. There are two main mechanisms that attempt to explain the involvement of cerebral vasculopathy in the course of the disease. The first mechanism is mycotic aneurysm rupture, which is most likely a result of an embolic event from brucella endocarditis. The second is arteritis, which results in lacunar infarcts, microhemorrhages, or venous thrombosis [13]. A total of seven cases of CVST secondary to neurobrucellosis have been reported in the literature [3,4,7-11]. CVST secondary to neurobrucellosis seems to have a female predominance (1:7; M:F), with a median age of 26.5 years ranging from 3 years to 52 years of age. Table 1 summarizes the demographic data, clinical features, investigations, and outcomes of previously reported cases. The most commonly reported symptoms were headache (62.5%) and fever (62.5%) followed by vomiting (50%). Lateralizing signs, such as weakness or urinary incontinence, were only seen in 25% of patients. A laboratory diagnosis of brucellosis can be achieved in different ways. The first method to achieve the diagnosis is by obtaining a blood, bone marrow, or other body fluid culture. In addition, serological tests, such as serum agglutination and enzyme-linked immunosorbent assays (ELISA), could be used to diagnose brucellosis [14]. Moreover, a neurobrucellosis diagnosis can be obtained by one of the following criteria: neurological symptoms, a positive CSF culture, a positive titer of antibodies targeting brucella in the CSF, lymphocytosis with high protein levels and low glucose levels in the CSF, and imaging findings [15]. The diagnosis of neurobrucellosis was reached in our patient through brucellosis growth in the blood culture and evidence of serum brucellosis antibodies accompanied by neurological symptoms. Furthermore, most of the cases in the literature were diagnosed using blood cultures and serum agglutination tests. Leukocytic pleocytosis was seen in most patients. Although interpreting these results should take the entire course of the patient. In our patient, the decision was made to promptly start antibiotic therapy due to the severity and hyperacuity of the presentation before sampling the CSF. Hence, no bacterial growth was observed in the cultures taken from the CSF samples. Although starting the antibiotic therapy might have altered the results of the CSF cultures, starting antibiotics in critical patients should not be delayed just to obtain the CSF samples. Hence, it is essential to take the patient’s history, clinical presentations, and all forms of investigations to promptly diagnose and accurately treat patients. The treatment of neurobrucellosis relies on antibiotics that can cross the blood-brain barrier [16]. However, the duration of the treatment varies depending on the time until clinical signs and symptoms and the CSF parameters are normalized [16]. The most favorable regimens are two or three antibiotics such as doxycycline, rifampin, ceftriaxone, and trimethoprim-sulfamethoxazole [16]. The average length of antibiotic therapy in the literature is 16 ± 10.5 weeks, ranging from 6 weeks to 36 weeks. All reported cases had complete resolution by the end of their treatment plan without any reported disabilities.

**Table 1:** Summary of patients' demographics, clinical presentations, thrombosis locations, and outcomes in the literature

Number	Author	Country	Age/sex	Headache	Fever	Nausea/ Vomiting	Other symptoms	Thrombosis location	Outcome
1	Zaidan and Al Tahan 1999 [7]	Saudi Arabia	23/ Female	Yes	No	Yes	Neck pain, photophobia, neck stiffness, and papilledema	Sagittal sinus	Complete resolution
2	Faraji et al. 2013 [8]	Iran	33/ Female	Yes	Yes	No	Seizures and night sweats	Left transverse sinus	Complete resolution
3	Lima JI et al. 2016 [3]	Portugal	30/ Female	Yes	No	Yes	Cough and coryza	Right lateral sinus, sigmoid sinus, and internal jugular vein	Complete resolution
4	Sakka et al. 2016 [4]	Tunisia	52/ Female	Yes	Yes	Yes	Neck stiffness and attention deficit	Right lateral transverse sinus and right internal jugular vein	Complete resolution
5	Ibrahimagić et al. 2017 [9]	Bosnia and Herzegovina	49/ Female	Yes	Yes	Yes	Papilledema, weakness, decrease in appetite	Left transverse sinus and left sigmoid sinus	Complete resolution
6	Turel et al. 2019 [10]	Turkey	6/ Female	No	No	Yes	Urinary incontinence, difficulty walking, and a decrease in the level of consciousness	Superior sagittal sinus	Complete resolution
7	Chaudhary et al. 2019 [11]	India	3/ Female	No	Yes	No	Seizures	Left transverse sinus	Complete resolution
8	This case	Saudi Arabia	23/ Male	Yes	Yes	No	Seizures and odynophagia	Left transverse sinus, sigmoid sinus, and left internal jugular vein	Complete resolution

## Conclusions

Neurobrucellosis is a rare cause of cerebral venous sinus thrombosis. It is imperative to keep neurobrucellosis as part of the differential diagnosis when treating patients diagnosed with cerebral venous sinus thrombosis, especially when treating individuals living in endemic areas. Cerebral venous sinus thrombosis secondary to neurobrucellosis has a favorable prognosis when an accurate diagnosis is made.
